# Inhibitory properties of a latent inhibitor after its compound preexposure with several novel stimuli: evidence from human conditioning

**DOI:** 10.3389/fpsyg.2025.1508789

**Published:** 2025-02-05

**Authors:** Unai Liberal, Gabriel Rodríguez, Paula Nogueiras, James Byron Nelson, Geoffrey Hall

**Affiliations:** ^1^Departamento de Procesos Psicológicos Básicos y su Desarrollo, University of Basque Country (UPV/EHU), Donostia-San Sebastian, Spain; ^2^Department of Psychology, University of York, York, United Kingdom; ^3^Department of Psychology, University of New South Wales, Kensington, NSW, Australia

**Keywords:** latent inhibition, inhibitory learning, nonreinforced preexposure, exposure therapy, comparative psychology

## Abstract

Latent inhibition refers to the retardation in learning an association between a target stimulus and an outcome when the target stimulus has been pre-exposed in the absence of consequences. The inhibitory properties of latent inhibitors have been the subject of controversy, as standard latent inhibition training—consisting of pre-exposure to the target stimulus alone—does not provide the latent inhibitor with the ability to pass a summation test, a key criterion for demonstrating genuine inhibition. However, previous research with animals has shown that a specific pre-exposure procedure, in which the target stimulus (A) is presented in compound with successive novel stimuli (An1, An2, An3…), can endow the target with sufficient inhibitory properties to pass both retardation and summation tests. To examine whether this phenomenon generalizes to humans, we conducted two experiments using a video game-based conditioning paradigm. Experiment 1 demonstrated that this compound pre-exposure schedule enhanced the retardation effect compared to standard pre-exposure or control conditions. Experiment 2 revealed that the target stimulus pre-exposed with novel stimuli significantly reduced responding when tested in compound with an excitatory conditioned stimulus, thus passing the summation test. These results suggest that compound pre-exposure facilitates the acquisition of inhibitory properties in humans, consistent with findings from animal studies. The findings are discussed within the framework of Hall-Rodríguez model, emphasizing the role of expectancy violation during pre-exposure in strengthening inhibitory associations. Implications for clinical applications, particularly in enhancing exposure therapy for anxiety disorders, are also considered.

## Introduction

Latent inhibition is operationally defined as a retardation in the acquisition of the conditioned response (CR) observed when the CS is exposed in the absence of reinforcement prior to the pairings between the conditioned stimulus (CS) and the unconditioned stimulus (US). Although a CS trained under these conditions is often referred to as a “latent inhibitor,” there are grounds to consider that it actually does not possess genuine inhibitory properties as those as manifested by a conditioned inhibitor (e.g., Rescorla, 1971; for a recent discussion on this topic, see Liberal et al., 2020). According to contemporary associative theory (e.g., Pearce and Hall, 1980; Rescorla and Wagner, 1972), the distinguishing quality of a conditioned inhibitor lies in its ability to suppress the otherwise activation of a mental representation of the US. It has been commonly accepted that for this inhibitory property to be demonstrated, the stimulus under consideration must pass two tests: a retardation test and a summation test (e.g., Rescorla, 1969; Sosa, 2022; Sosa and Ramírez, 2019; but see Papini and Bitterman, 1993; Savastano et al., 1999 for an alternative view about the conditions required to detect inhibition). In applying this double empirical criterion, a fundamental distinction emerges between a latent inhibitor and a conditioned inhibitor. Several studies have shown that although a latent inhibitor is capable of passing a retardation test, it has not been shown to be capable of passing a summation test (e.g., Liberal et al., 2020, 2022; Nelson et al., 2021; Reiss and Wagner, 1972; Rescorla, 1971; Solomon et al., 1974; cf., Kremer, 1972).

More specifically, in a retardation test, the target stimulus, with presumed inhibitory properties, is paired with a US over several trials. Those pairings should endow the CS with excitatory properties through which it can “excite” a representation of the US, resulting in a CR. If the target CS begins the conditioning training already possessing inhibitory properties, the manifestation of its excitatory properties in the form of a CR will be retarded in comparison to control conditions where the CS starts associatively neutral. Following the operational definition of latent inhibition, a latent inhibitor is a stimulus that passes the retardation test. However, by itself, this retardation is not sufficient evidence that latent inhibitors have genuine inhibitory properties. Non-reinforced pre-exposure could result in inattention to the stimulus that impairs its subsequent conditioning (e.g., Hall and Rodríguez, 2010; Lubow, 1989; Mackintosh, 1975; Pearce and Hall, 1980). There is good evidence that this attentional learning exists (e.g., Rescorla, 1969; Rodríguez et al., 2019), which makes it necessary to resort to the use of a summation test.

In a summation test, potential inhibitory properties are tested by presenting the target stimulus in compound with a transfer CS that has been paired earlier, and independently, with the US. The logic of this test is that the transfer CS will activate the US representation but that, if the target possesses inhibitory properties, it will counteract such activation and reduce any potential resulting CR. It is known that different training procedures in which the target is presented explicitly uncorrelated with the US, succeed in endowing stimuli with properties that allow them to pass both the retardation and summation tests (Laing et al., 2021; Sosa, 2022; Sosa and Ramírez, 2019). However, this is not the case for the simplest non-reinforced pre-exposure training in which the target stimulus is initially presented repeatedly alone and in the absence of any consequence, including any US. This sort of pre-exposure training seems to endow the target stimulus only with the ability to pass the retardation test (Liberal et al., 2020, 2022; Nelson et al., 2021; Reiss and Wagner, 1972; Rescorla, 1971; Solomon et al., 1974; Wagner and Rescorla, 1972; cf., Kremer, 1972). That is, a latent inhibitor is no more effective than a novel stimulus in interfering with the manifestation of a CR in the presence of an excitatory transfer stimulus. This failure casts doubt on whether non-reinforced pre-exposure endows stimuli with genuine inhibitory properties and points to the fact that the main effect of such pre-exposure is a reduction in attention to the stimulus.

However, our recent results have led us to reconsider the relationship between latent inhibition and its net inhibitory properties as assessed by a summation test. In several experiments using appetitive and aversive conditioning procedures (Liberal et al., 2020, 2022), with rats as subjects, we have shown that a latent inhibitor can indeed pass a summation test, depending on the conditions present during pre-exposure. Specifically, these conditions consist of pre-exposing the target stimulus, A, in combination with several novel stimuli in the absence of an outcome (An1, An2, An3, An4…).

Those experiments were driven by the theory presented by Hall and Rodríguez (2010) on the learning that takes place during non-reinforced pre-exposure to stimuli. This theory assumes that a novel stimulus is capable of activating with its presentation the expectation that something may occur. Disconfirmation of this expectation during non-reinforced pre-exposure would lead to the actual processing of the “absence of consequences” and would allow the representation of the stimulus to be gradually associated with the representation of that absence of consequences. Under conditions in which the stimulus (e.g., A) is presented alone, the theory predicts that the strength of the association between the stimulus and the absence of consequences will allow the initial expectation that something may occur to be neutralized (for a more formal exposition of the model predictions, with computational simulations, see Liberal et al., 2020). That is, the magnitude of the strength of the association between the stimulus and the absence of consequences is expected to equal, but never exceed, the strength of the initial preset association that triggers the expectation that something might happen. Under these conditions, the target stimulus would not be able to pass the summation test after its non-reinforced pre-exposure, not having acquired net inhibitory properties.

But, interestingly, the theory predicts that under certain conditions, a stimulus pre-exposed in the absence of consequences could indeed acquire an association with the absence of consequences whose magnitude exceeds the strength of the initial excitatory association. Specifically, a training that would be capable of generating this situation would be one in which the target stimulus (A) is presented in the absence of consequences but accompanied by successive novel stimuli (An1, An2, An3…). The continuous presence of a novel stimulus each time A is presented would ensure a high activation of the expectation that something is going to happen and, consequently, effective processing of the absence of consequences once that expectation is subsequently disconfirmed. This would allow the target stimulus A to establish an association with the absence of consequences of greater magnitude than its preestablished excitatory association, such that its presentation would evoke the expectation that nothing is going to happen. This evocation would be what allows the pre-exposed target stimulus under these particular conditions to pass the summation test (Liberal et al., 2020, 2022).

These findings may have important clinical implications because of the role of conditioned inhibitors in safety learning (Craske et al., 2014, 2022; Laing and Harrison, 2021; Laing et al., 2021; Odriozola and Gee, 2021). The standard training used to model safety learning in the laboratory is inhibitory conditioning (e.g., Sosa and Ramírez, 2019), and requires the explicit presentation of aversive USs. For example, the most commonly used conditioned inhibition training involves the intermixed presentation of two types of trials: reinforced trials in which a non-target stimulus (the exciter) is followed by the occurrence of the aversive event or US (X → US) intermixed among non-reinforced trials in which × is presented simultaneously with the target stimulus, A, but not followed by consequences (AX → no event). This type of training turns stimulus A into a conditioned inhibitor, endowing it with the ability to interfere with fearful and/or anxious CRs generated by X.

Conditioned inhibitors are fairly specific to their outcomes. That is, if stimulus A was trained as a conditioned inhibitor for shock, it has no effect on a stimulus predicting food (Rescorla and Holland, 1977; Holland, 1989; see also Dickinson and Dearing, 1979 for further discussion of inhibition’s interactions with motivational systems). Latent inhibitors, however, appear more general. Pre-exposure to a stimulus retards conditioning regardless of the outcome used (Best, 1975; Killcross and Balleine, 1996; Rescorla, 1971). Thus, it would be expected that a stimulus pre-exposed in the presence of several other co-occurring stimuli that acquires the ability to pass a summation test might function as a more general type of safety signal.

Our previous work Liberal et al. (2020, 2022) importantly suggests that it would be possible to endow a stimulus with similar conditioned inhibition-like properties (i.e., convert it into a safety cue) through its non-reinforced exposure. It would suffice to present the target stimulus in the company of several novel stimuli in the absence of any significant outcome. This procedure could have important implications for the development of new models of exposure therapy that would not require the presentation of aversive events. In order to determine whether our previous findings with rats generalize to humans, we tested the ability of our special pre-exposure schedule (An1, An2, An3…) in endowing inhibitory properties to A, in a paradigm whose effectiveness in generating a latent inhibition effect in humans has been previously demonstrated (Nelson et al., 2021, 2022).

**Figure 1:**
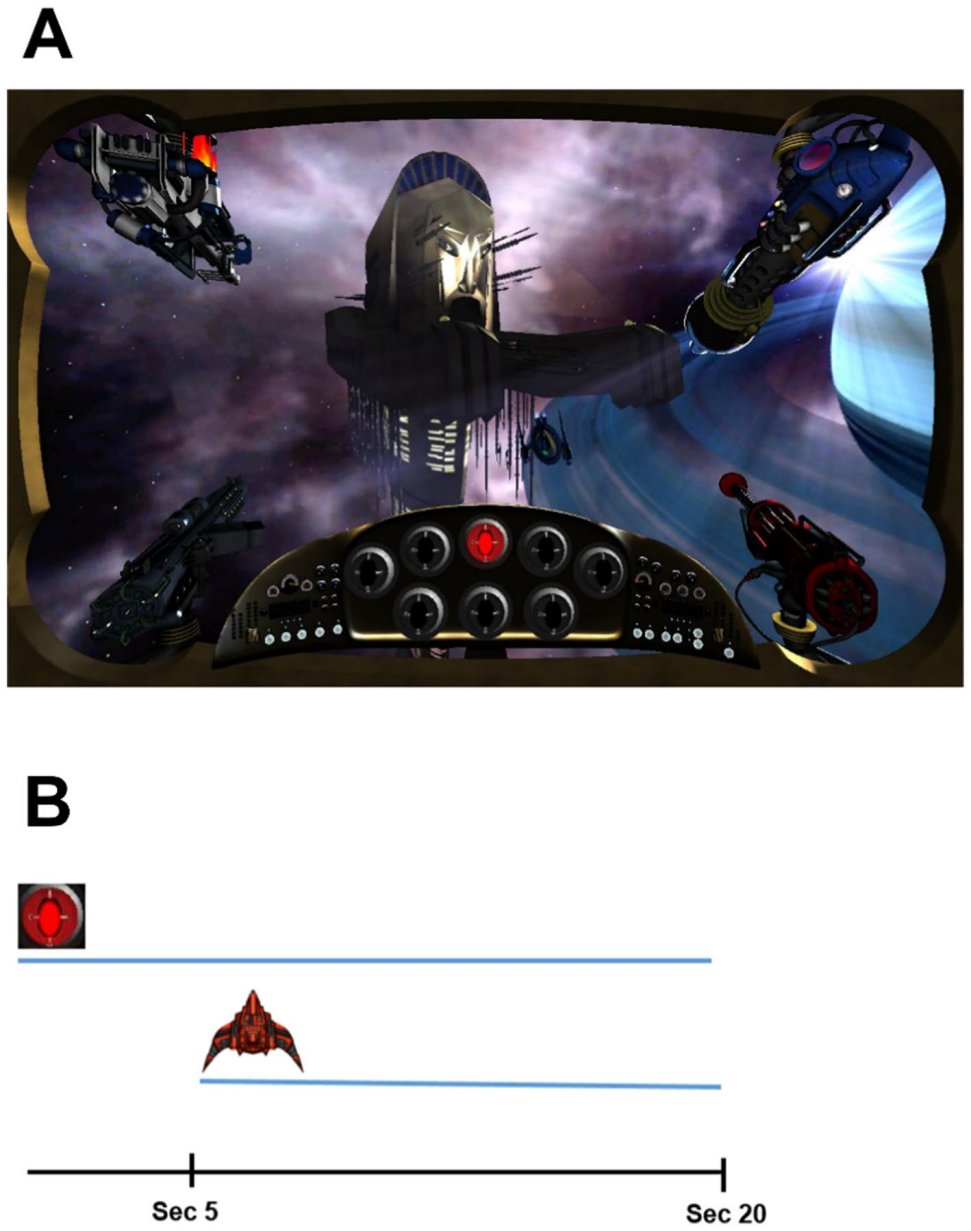
The experimental videogame apparatus. **(A)** First person view of the videogame during the experiment in which a red sensor is illuminated in the panel control. **(B)** Description of a conditioning trial: the sensor CS is illuminated for 20-s and the attack of the spaceship occurred after 5 s.

In this paradigm, the participant plays a video game in which he/she is asked to adopt the role of protecting space stations in galaxies from invaders. On the computer screen (see Figure 1A), participants see a first-person view of a space station in a galaxy background through the viewscreen of their spaceship. The US outcome is a specific enemy spaceship which attacks the station at various times (i.e., on several conditioning trials). CSs consist of the 5-s flashing of a light sensor, with a specific color, located on a panel in the viewscreen (see Nelson et al., 2014 for more images). Conditioning trials consist of the 20-s illumination of a sensor CS and the occurrence of a spaceship attack after the first 5 s that persists for 15 s, with the spaceship existing the scene with the termination of the sensor (see Figure 1B).

The player/participant can repel the enemy ship’s attack by activating a specific weapon through repeated pressing of a key on the computer keyboard. In order for the weapon to be active at the time of the enemy spaceship’s (the US’s) appearance, the participant must “charge” the weapon a few seconds prior to the arrival of the spaceship. The most effective defense against the enemy ship requires anticipating its occurrence, signaled by a sensor, and beginning to respond (i.e., to charge the weapon) at the occurrence of the CS. In this method, non-reinforced stimulus pre-exposure consists of the turning on a certain colored sensor without the occurrence of a spaceship.

Exploiting this procedure, we designed two experiments, one including a retardation test (Experiment 1) and the other including a summation test (Experiment 2), to assess the possible inhibitory properties acquired by pre-exposing a target stimulus (the lighting of a sensor) simultaneously together with other non-target stimuli (the lightening of other sensors: n1, n2, n3…), in the absence of reinforcement. We expected to replicate our findings using non-human conditioning procedures, finding the target stimulus A is able to pass both the retardation and summation tests.

## Experiment 1

The first experiment was the retardation test (see Table 1). All participants received the same conditioning training during the second phase of the experiment in which the target CS A (the illumination of a red sensor) signaled the occurrence of the US (the invader spaceship). There were four groups differing in the treatment received during the initial preexposure phase. One group was a standard latent-inhibition condition (group A), in which participants, prior to conditioning, received 7 non-reinforced presentations of the target stimulus A alone (A1, A2, …, A7). Another group (group AN) received the same number of presentations of the target stimulus A but always in compound with a novel visual stimulus (which consisted of the illumination of another sensor with a color different from red, changing across trials; An1, An2, …, An7). In addition, there were two control groups (N and NP) that had no experience with the target stimulus A prior to the conditioning phase. During the pre-exposure, participants from Group N received presentations to the same novel stimuli that were presented to the AN group (i.e., n1, n2, …, n7), but without the presence of the target stimulus A. Participants from group NP received exposure to the stations and background galaxy during the pre-exposure phase, without any exposure to a sensor.

**Table 1:** Experimental designs.

Experiment 1: retardation		
Group	Preexposure	Conditioning
AN	7 × AN (An^1^, An^2^, An^3^… …An^6^, An^7^)	8 × (A→US)
N	7 × N (n^1^, n^2^, n^3^… …n^6^, n^7^)	8 × (A→US)
A	7 × A (A, A, A… …A, A)	8 × (A→US)
NP		8 × (A→US)

Experiment 2: Summation			
Group	Preexposure	Conditioning	Test
AN	7 × AN (An^1^, An^2^, An^3^… …An^6^, An^7^)	10 × (X→US)	AX
N	7 × N (n^1^, n^2^, n^3^… …n^6^, n^7^)	10 × (X→US)	AX
A	7 × A (A, A, A… …A, A)	10 × (X→US)	AX
NP		10 × (X→US)	AX

Each letter represents a cue (the illumination of a sensor), the US consisted of the apparition of an invader spaceship.

We expected to observe results similar to those obtained in our previous experiments with rats with conceptually similar designs (Liberal et al., 2020, 2022). We should observe retardation in conditioning (i.e., a latent inhibition effect) in the groups receiving pre-exposure to the target stimulus A (the AN and A groups) relative to the control groups (the N and NP groups). The presence of the novel sensor on each pre-exposure trial in group AN should continue to generate the expectation that something might occur, which does not occur, producing a particularly strong A → no event association in that group.

### Methods

#### Participants

The sample size of this experiment was estimated on the basis of the sizes of the latent inhibition effects ( $\eta_{p}^{2}$ = 0.23) detected in previous studies (e.g., Nelson et al., 2022) using the same procedure, and similar parameters, to those used here. A sample-size of 56 participants (14 in each of the groups), gave us a power of 0.78 to detect an effect such as that reported by Nelson et al. (2022). Participants were students from the University of the Basque Country (36 females; mean age 20.2 years) who agreed to participate after being informed that the experiment would involve a learning task. All participants had normal or corrected-to-normal vision. They were randomly assigned to one of the four groups. The experimental protocol was approved by the Research Ethics Committee of the University of the Basque Country for the Investigation with Human Beings (CEISH).

#### Apparatus and stimuli

The experiment was run on PCs with the screen resolution set at 1280 × 800 pixels on 22-in monitors. All participants used headphones to listen to the music and auditory stimuli from the videogame. The videogame employed was that developed by Nelson et al. (2014); all the details and visuals of the methodology not described below can be found there (the videogame can be downloaded in http://drjbn.wordpress.com/the-learning-game-download-links/, where the present materials and future updates can be found). In this 3D videogame, the participants/players have a view from inside a spaceship through a viewscreen. In the present experiment, two backgrounds were used: a response-training background and a station/galaxy environment in which the pre-exposure and conditioning phases took place. In the training environment the participant is taught to emit the response of charge the ship’s weapons. In this environment, the participants view is as if their ship was inside of a large green wireframe gridded cube. The pre-exposure and conditioning phases took place in a different environment, the “Boutonia” galaxy. In this galaxy, the participant views a large rotating space station in the shape similar to that of a sphinx, stars and a large blue planet to the right of the station. Light radiating from a sun behind the planet can also be seen.

Near the bottom of the screen view (see Figure 1), there is a control panel inside the spaceship. The panel consists of two rows of sensors. Each sensor is shaped like an oval disc and can be lit with different colors and intensity (by adjusting the RGB parameters, min/max 0–255), with an on/off flashing at a rate of three cycles per second. On the top row of the control panel there are 5 sensors and on the bottom row three sensors. The diameter of each sensor is 50 pixels when illuminated.

In the present experiment, the target stimulus A was the illumination of the central sensor in the top row, which emitted a low-intensity red light (RGB = 32, 0, 0). The novel stimuli (N) consisted of switching on other sensors with lights of other colors, namely: a green light (RGB = 0, 255, 0) on the second sensor from the left of the top row, a blue light (RGB = 0, 0, 255) on the third sensor from the left of the top row, a purple light (RGB = 255, 0, 255) on the second sensor from the right of the top row, a pink light (RGB = 59, 154, 135) on the second sensor on the left of the top row, a brown light (RGB = 146, 102, 77) on the right sensor of the bottom row, an orange light (RGB = 255, 94, 0) on the third sensor on the right of the top row, and a cyan light (RGB = 114, 208, 246) on the right sensor of the bottom row.

In the videogame, there are four spaceships that could be used as a US. All four were used during the response-training phase, in which the participants were taught how to fire the ship’s weapons. Only one of them was subsequently used a US in the experiment, the so-called “Stellarian” in the game’s cover story. The “Stellarian” was an off-white colored ship that emerged from the top left of the screen, and was repelled by a weapon on the top left of the screen that fired fireballs, called “Extinction Fire.” The weapon was activated by pressing the left tab key on the keyboard. Once 5 s of key presses at a rate of 3 s were accumulated, the weapon activated and began firing at the attacking spaceship, as long as the rate was maintained and the spaceship was present.

#### Procedure

##### Instructions and response-training phase

Participants were told (through a text panel in the screen and by audio voice) that they would protect space stations from attack by invaders and that they must learn how to use the weapons to do so. For each invader spaceship, participants were instructed that something was about to appear, and then the ship flies into the screen. Participants were told the name of the ship, which weapon to use to repel it, and which key activated the weapon. They were instructed to press the key rapidly and repeatedly until the weapon was firing and the invader had fled. There were two consecutive trials with each of the four spaceships, with the order of the ships appearance randomly determined. After the last response training trial, participants were told that they were ready for patrol.

Instructions encouraged participants to have weapons ready when they thought that invaders were going to appear, so that they might attack the invader upon its arrival, before it attacked the space station. They were also informed that invaders might never appear and participants could enjoy “the beauty of the galaxies and music beamed from the stations” while patrolling. Participants were then told to protect the “Boutonians” and were transported to the galaxy described earlier. No information about the sensors was provided.

##### Preexposure phase

All the groups received 7 pre-exposure trials, with a variable inter-trial interval (ITI) averaging 20s. For group AN, on those seven trials, the target sensor A was illuminated for 20 s at the same time as one of the N sensors was also lit. The N group received the same lighting of the N-sensors as that received by the AN group, but without the occurrence of the lighting of the target A-sensor. There were seven different N-sensors, so that there was always a new stimulus in each trial. In both groups AN and N, the lighting of the N-sensors occurred in the following order: green, blue, purple, pink, brown, orange and cyan. Group A received the same number of lightings of sensor A as group AN, but this lighting was always presented alone, with no concurrent lighting of other sensors. In the NP group, no sensor was illuminated during pre-exposure.

##### Conditioning

Conditioning began 20 s after the last pre-exposure trial, without any explicit indication to the participants. All groups received eight trials in which the 20 s illumination of the red sensor was paired with an attack from the Stellarian. The Stellarian appeared after 5 s of sensor illumination and remained for 15 s regardless of the participant’s behavior. The random ITI averaged 20 s.

#### Data treatment and analysis

The number of times that participants pressed the left tab key (assigned to the weapon that repelled the Stellarian) was recorded during each second for the 5 s prior to illumination of the sensor(s) (pre-CS period), as well as in each of the 20 s in which illumination occurred (CS period). We constructed difference scores where the responding during the pre-CS was subtracted from the CS to adjust for the influence that any unconditioned tendency to respond might have across individuals.

Data were analyzed with ANOVA or, where appropriate, *t-*tests or Duncan pairwise mean comparison tests were used. Pre-CS data and responses to the CS prior to conditioning were skewed and contained numerous zeros. Those data were analyzed with non-parametric tests. A statistical significance criterion of *p* < 0.05 was adopted. Effect sizes for ANOVAs are reported as partial eta squared ( $\eta_{p}^{2}$ ), and those for pairwise comparisons are reported using Cohen’s *d*. The 95% confidence intervals (CI) around the effect sizes are also reported in parentheses following the effect size and calculated by software provided by Nelson (2016).

### Results and discussion

In the preexposure phase, average responding during the pre-CSs periods was very low, the mean responses per second being 0.04, 0.05, 0.04, and 0.06, for the groups AN, N, A and NP, respectively. The overall level of response during the CS-periods in the preexposure was also quite low, the mean responses per second being 0.16, 0.13, 0.01, and 0.03, for the groups AN, N, A and NP, respectively. There were no group differences with either measure, *p*s > 0.357.

In the conditioning phase, key pressing during the pre-CS periods still remained quite low, the mean responses per second being 0.17, 0.43, 0.32, and 0.28, for groups AN, N, A and NP, respectively, and did not differ between the groups, *p* > 0.214.

Responding during the CS was lower in this experiment than in previous experiments using this method (c.f., Nelson et al., 2021). The difference could be simple differences between samples, or the arbitrary use of the Stellarian as an outcome. The weapon used against the Stellarian required the use of the left hand to operate the left tab key. With the majority of the population being right-handed, the lower rates could reflect the handedness of the sample. Also, the red light used was dimmer than that used in prior experiments, which might also contribute to the difference. Given that responding was low, it might be unduly influenced by levels of pre-CS responding. For that, we constructed difference scores where the responding during the pre-CS was subtracted from the CS to adjust for the influence that any unconditioned tendency to respond might have across individuals.

**Figure 2:**
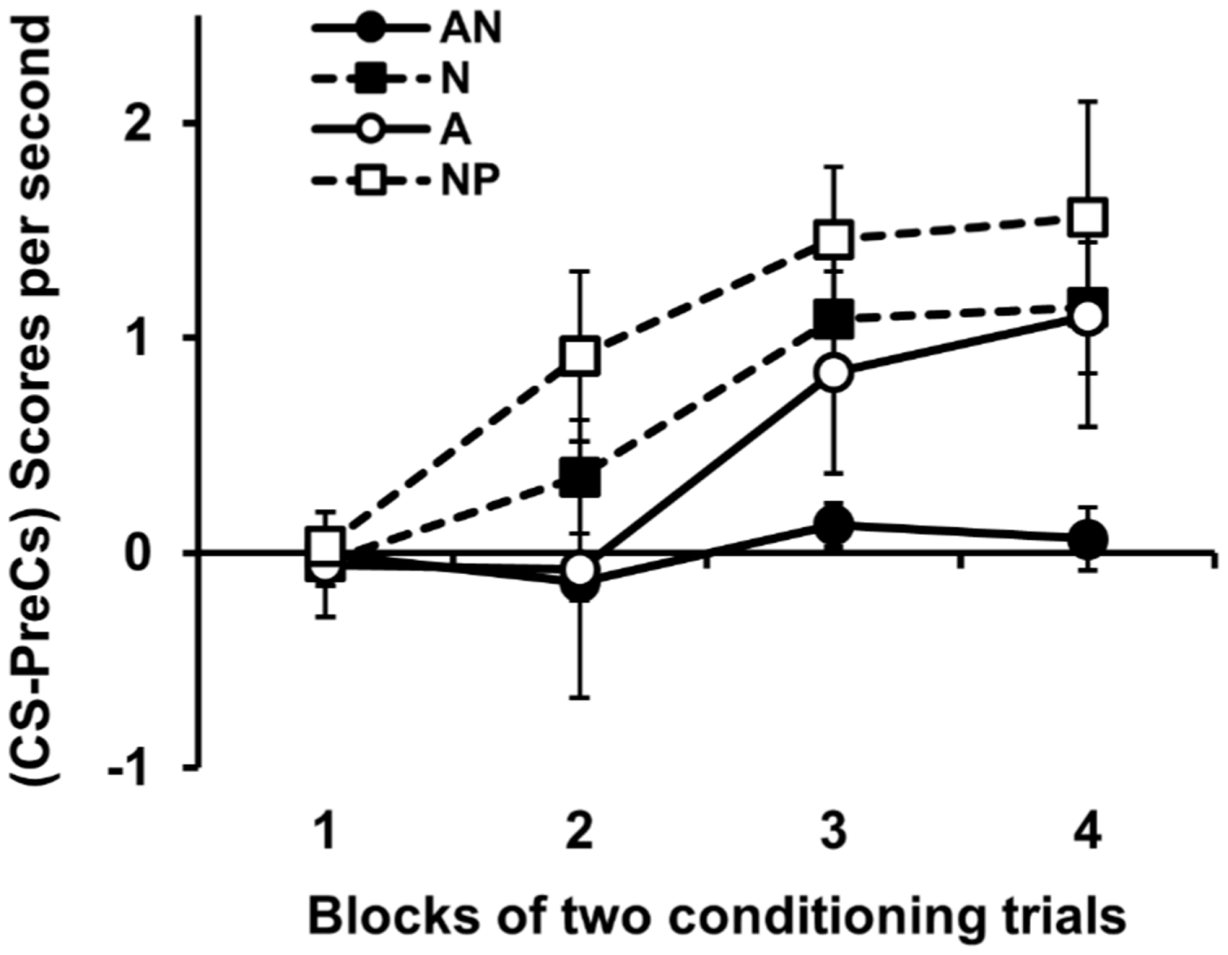
Results of experiment 1. The left panel shows the group mean scores (CS-PreCS responses) per second (+/− SEMs) during the conditioning phase in Experiment 1. The group AN received prior exposure to A in compound with a novel stimulus on each trial, the group N received presentations of those novel stimuli alone, the group A received exposure to A alone, and the control group NP received exposure to the experimental context. The right panel shows the group mean scores (CS-PreCS responses) per second (+/− SEMs) to the target stimulus A during its conditioning.

Figure 2 shows the most important results of the experiment, the group-mean difference scores (CS-Pre-CS) over the course of the 4 blocks of two conditioning trials. Difference scores clearly increased in the NP group throughout the conditioning training. The AN, A and N groups showed, however, retarded acquisition of this increase in responding to the CS. The retardation was moderate in the case of groups A and N in which the level of response to the CS eventually increased with the course of training. However, the retardation shown by the group AN was more profound with no evidence of a substantial increase in the level of response after the 4 blocks of training. An ANOVA 4 (Group) × 4 (Block of trial) conducted on these data revealed a significant effect of Group, *F*(3, 52) = 4.39, *p* = 0.008, $\eta_{p}^{2}$ = 0.202, 95% CI = [0.02–0.35]. *Post hoc* pairwise comparisons using Duncan’s test revealed that the overall level of responding showed by group NP was higher than that showed by groups AN and A. The ANOVA also showed a significant effect of Block, *F*(3, 156) = 15.75, *p* < 0.0001, $\eta_{p}^{2}$ = 0.23, 95% CI = [0.11–0.32]; the interaction Group × Block was also a low-probability result, *F*(9, 156) = 1.79, *p* = 0.073, $\eta_{p}^{2}$ = 0.09, 95% CI = [0.00–0.13]. On the first block of training there were no differences among groups, *F*(3, 52) = 0.11, *p* = 0.957. Differences became significant on Block 2, *F*(3, 52) = 3.91, *p* = 0.014, $\eta_{p}^{2}$ = 0.18, 95% CI = [0.01–0.33], and Block 3, *F*(3, 52) = 3.31, *p* = 0.027, $\eta_{p}^{2}$ = 0.16, 95% CI = [0.00–0.3], and were close to the significance level on Block 4, *F*(3, 52) = 2.7, *p* = 0.055, $\eta_{p}^{2}$ = 0.13, 95% CI = [0.00–0.27]. Subsequent pairwise comparisons using Duncan’s test showed that, on Block 2, group NP showed greater responding to the CS than groups AN, N and A. These differences indicate a latent inhibition effect in all three groups pre-exposed to some type of sensor illumination, both the groups exposed to the target sensor A (Groups AN and A) and the group exposed to the set of sensors of color other than A (Group N). Pairwise comparisons using Duncan’s test with data from Blocks 3 and 4 showed that the AN group responded less than the NP Group, supporting the idea that in the AN group the latent inhibition effect was more profound.

The results of the present experiment partially confirmed our predictions. As expected, a latent inhibition effect was observed in the two conditions of exposure to the target stimulus A (groups AN and A). A generalization effect of latent inhibition was also observed, as conditioning to A was somewhat retarded after pre-exposure to the “n” set of non-target sensors (group N). More unexpected were the magnitudes of some of the observed effects. On the one hand, we expected to have observed a stronger latent inhibition effect in Group A. The relatively small effect observed in this group could have been due to the low intensity of the target stimulus (e.g., Rodríguez and Alonso, 2002; Rodríguez et al., 2015). We decided to use a low-intensity target CS because in previous work with rats we have found that the effects of compound pre-exposure to a target stimulus are maximized when the target has a relatively low intensity compared to the accompanying stimulus or stimuli (e.g., Rodríguez and Hall, 2008; Hall and Rodríguez, 2011; Rodríguez et al., 2014). Indeed, the results from Group AN are consistent with this idea and might constitute a demonstration in humans of the potentiation of latent inhibition effect found by Rodríguez and Hall (2008) in appetitive and aversive conditioning procedures in rats (see also Leung et al., 2011, 2013). In this effect, the retardation in conditioning observed after non-reinforced pre-exposure to the CS is enhanced when that stimulus is pre-exposed in compound with another more intense stimulus. In the present experiment, the target CS (the low-intensity sensor A) was pre-exposed not with one, but with several stimuli more intense than it (the N-sensors), which could have led to such a potentiation of the latent inhibition effect. While this aspect of the results is interesting in itself, what interests us more in the present study whether the pre-exposure schedule received by the Group AN endows the target stimulus with the ability to not only pass a retardation test but also to pass a summation test. We examined this possibility in the following experiment.

## Experiment 2

In this experiment we conducted a summation test (see Table 1). All participants received the same conditioning and test training during the second and third phases of the experiment. During conditioning, a non-target CS × (the illumination of a yellow sensor) signaled the occurrence of the US (the invader spaceship). After this conditioning phase, the ability of the target stimulus A to interfere with the CR evoked by × was assessed in non-reinforced test trials with × and A presented in compound. There were four groups that differed in the treatment received during the initial pre-exposure phase in the same way as in Experiment 1 (groups AN, N, A, and NP). We expected to find a similar pattern of results to that observed in our previous experiments using conditioning techniques with rats. That is, we expected to find clear evidence of a summation effect in Group AN, which would indicate that the program of preexposure received by this group endowed the target stimulus A with inhibitory properties capable of interfering with the excitatory properties of × in the test.

### Methods

The participants were 60 volunteer students from the University of the Basque Country (36 females; mean age = 23.7 years) that were randomly assigned to one of four groups (*n* = 15). All the details of the apparatus and the procedure not specified here were the same as those described for Experiment 1.

The initial response training and the preexposure phases were conducted in the same manner as in the previous experiment. Afterwards, all subjects received 10 trials of conditioning with a new CS X, in which the 20s illumination of a yellow sensor (RGB = 255, 255, 0) presented in the middle disc of the lower row was followed by an attack from the Stellarian spaceship appearing from the upper left quadrant of the screen. The summation test began 20s after the last conditioning trial. For all groups, there were 2 non-reinforced trials in which the conditioned sensor CS × (the yellow light) and the target sensor A (red) were illuminated simultaneously for 20s, but the spaceship did not appear.

### Results and discussion

The overall response level during the CS-periods in the preexposure phase were: 0.1, 0.1, 0.16 and 0.01, for groups AN, N, A and NP, respectively.

**Figure 3:**
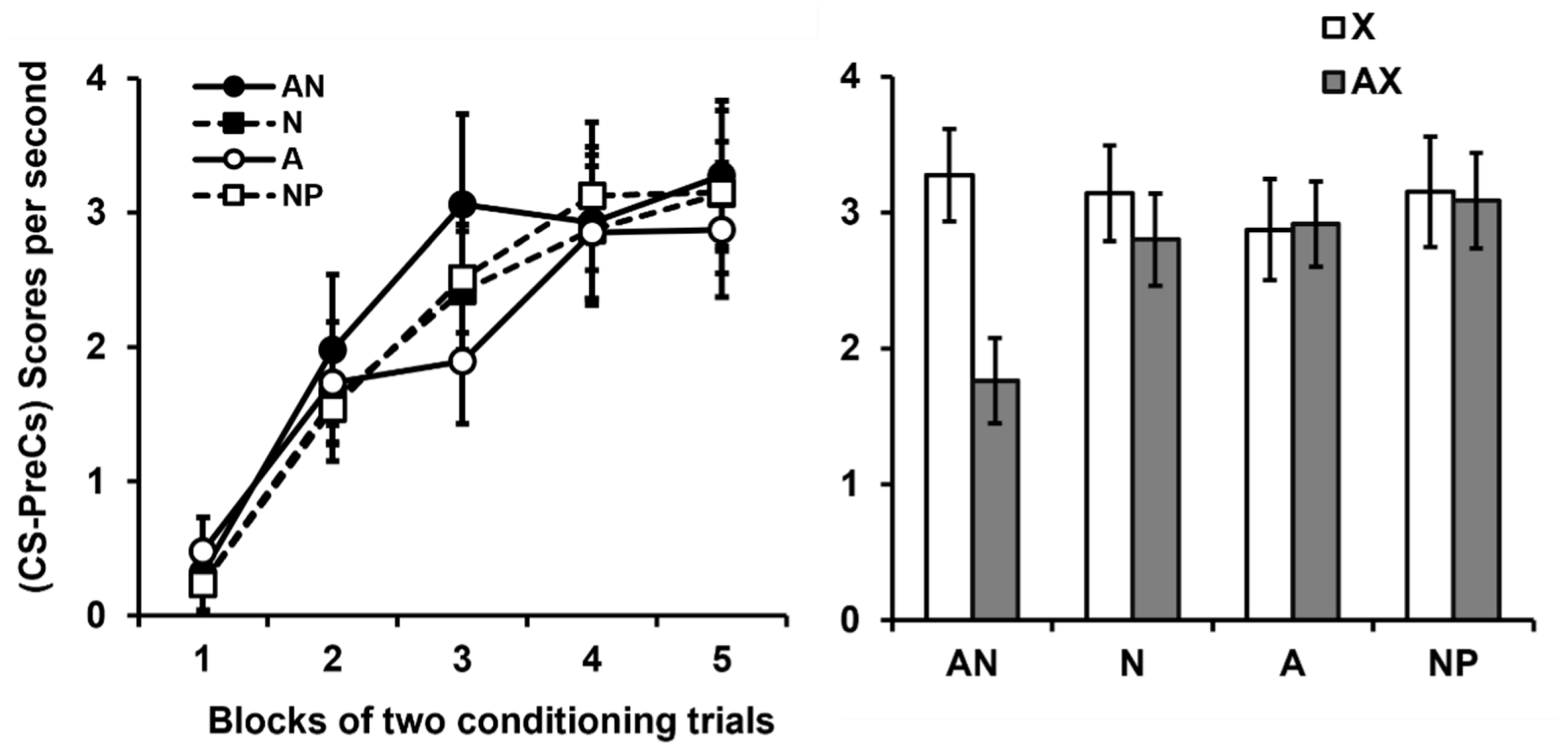
Results of experiment 2. Note. Group mean scores (CS-PreCS responses) per second (+/− SEMs) to the non-target stimulus × during its conditioning (Left panel). Group mean scores (CS-PreCS responses) per second (+/− SEMs) to the non-target stimulus × during the last block of conditioning trials and to the AX compound during the block of test trials (Right panel).

The mean responses per second during the pre-CS periods of the conditioning phase were 0.24, 0.46, 0.37 and 0.28, for the groups AN, N, A and NP, respectively. And the mean responses per second during the pre-CS periods of the test phase were 0.34, 0.42, 0.92 and 0.18, for the groups AN, N, A and NP, respectively. There were no group differences with either measure, *p*s > 0.224. The panel of Figure 3 shows the group-mean difference scores (CS-Pre-CS) over the course of the 5 blocks of two conditioning trials with the transfer stimulus Y. Scores progressively increased, apparently at similar rates in all the groups, across the blocks of trials. An ANOVA 4 (Group) × 5 (Block of trial) conducted on these data confirmed this impression, revealing that only the effect of Block was significant, *F*(4, 224) = 59.88, *p* = 0.0001, $\eta_{p}^{2}$ = 0.58, 95% CI = [0.42–0.58]. Neither the effect of Group, *F*(3, 56) = 1.25, *p* = 0.297, nor the interaction Group × Block were significant, *F*(12, 224) = 1.09, *p* = 0.365.

We analyzed the ability of stimulus A to inhibit the response evoked by CS × by comparing the response to × shown by participants on the last block of two conditioning trials with that exhibited to AX on the block of two test trials. The panel of Figure 3 shows this comparison. It can be seen that the groups not pre-exposed to the target stimulus A (Groups N and NP) showed a level of response to × at the end of conditioning fairly similar to that showed to AX at testing. The group exposed to the target stimulus alone (Group A) showed same responding levels to AX and × at the end of conditioning. However, the group that received the special pre-exposure of interest (pre-exposure to the target stimulus in the company of a novel stimulus on each trial, Group AN) showed a much more marked reduction in responding to AX. This would be indicating that, during this type of pre-exposure, the target stimulus A acquired properties that allowed it to inhibit subsequently the CR to X. A 4 (Group) × 2 (Stimulus × or AX) ANOVA conducted on these data revealed a significant main effect of Stimulus, *F*(1, 56) = 12.37, *p* = 0.001, $\eta_{p}^{2}$ = 0.18, 95% CI = [0.03–0.34]. The main effect of Group was not significant, *F*(3, 56) = 1.34, *p* = 0.303, but critically, the Stimulus × Group interaction reached the significance, *F*(3, 56) = 3.36, *p* = 0.025, $\eta_{p}^{2}$ = 0.15, 95% CI = [0.01–0.29]. A series of additional analyses were performed in order to clarify the source of this interaction. One way-way ANOVAs were used to assess the main effect of Group on responses to × and AX. These analyses showed that the groups did not significantly differ in responding either to X, *F*(3, 56) = 0.83, *p* = 0.478, or AX, *F*(3, 56) = 2.04, *p* = 0.118. On the other hand, the more powerful within-subjects comparisons of the responses to × and AX only showed a significant reduction in responding to AX in group AN, *t*(14) = 4.01, *p* = 0.001, *d* = 1.03, 95% CI = [0.39–1.65], as assessed by a paired samples *t*-test. No significant differences in this regard were observed in groups N, *t*(14) = 1.72, *p* = 0.107, A, *t*(14) = 1.41, *p* = 0.188, or NP, *t*(14) = −0.28, *p* = 0.783.

In the literature (e.g., Rescorla, 1971; see also, Liberal et al., 2020, 2022; Nelson et al., 2021), the comparison on the basis of which it had been concluded that a latent inhibitor fails the summation test was to compare a condition in which the target stimulus is exposed alone (equivalent to our Group A) with a control condition without pre-exposure (equivalent to our Group NP). We conducted a separate analysis of these two conditions included in our design. An ANOVA with Group (A or NP) and Stimulus (X or AX), revealed neither main effects of Group, *F*(1, 28) = 3.1, *p* = 0.089, nor Stimulus, *F*(1, 28) = 1.4, *p* = 0.245, nor the interaction between these two variables, *F*(1, 28) = 1.96, *p* = 0.173, were significant. These results, thus, replicate the usual result found in the literature in which a standard latent inhibitor fails to pass the summation test.

A similar analysis to the one just described but comparing our special latent inhibition training condition (Group AN) with the control NP group, revealed a different pattern of results. An ANOVA with Group (AN or NP) and Stimulus (X or AX) as variables revealed that the main effect Group was not significant, *F*(1, 28) = 1.24, *p* = 0.275. However, the main effect of Stimulus, *F*(1, 28) = 10.52, *p* = 0.003, $\eta_{p}^{2}$ = 0.27, 95% CI = [0.03–0.48] and, critically, the Group × Stimulus interaction was significant, *F*(1, 28) = 12.56, *p* = 0.001, $\eta_{p}^{2}$ = 0.31, 95% CI = [0.05–0.51]. Further analyses performed in order to clarify the source of this interaction showed that groups did not differ in their responding to × on the last block of trials of conditioning, *t*(28) = 0.06, *p* = 0.947. However, responding to AX was significantly less in Group AN than in Group NP, *t*(28) = 2.2, *p* = 0.036, *d* = 0.8, [0.05–1.54].

## General discussion

The present results constitute an extension to the human domain of the effects we have previously found using conditioning techniques with rats (Liberal et al., 2020, 2022). Non-reinforced pre-exposure of a target stimulus (A) in compound with several novel stimuli (An1, An2, An3…) endowed it with the ability to pass both the retardation (Experiment 1) and the summation (Experiment 2) tests. We interpret these results as evidence that this specific pre-exposure schedule confers on the pre-exposed stimulus genuine inhibitory properties. However, before accepting this conclusion, it is necessary to consider and rule out possible alternative explanations, especially those that allude to the influence of generalization and external inhibition on our most critical results, which are those of the summation test in Experiment 2.

With respect to generalization, a first alternative explanation could hold that what allowed the AN group to pass the summation test was some kind of additive effect. In this group, the effect generated by the experience with A could have been added to the generalization of the effect generated by the experience with the novel stimuli N (n1, n2, n3…). This hypothesis, however, faces some problems. The first is that it assumes that pre-exposure to A and pre-exposure to N stimuli independently generate net, additive inhibitory effects. For the explanation to gain traction, we would therefore need to find a mechanism (different from the one proposed by Hall and Rodríguez, 2010) that would be able to explain how these pre-exposure conditions generate such a net inhibitory value on the stimuli in those conditions. And, what may be more important, we would also need to find evidence that A passes the summation test in groups A and N, since in these groups the putative additive effects would have been generated separately. The consistent absence of such evidence in the results of our experiments (and in the literature) weakens this type of alternative explanation.

Another explanation based on the phenomenon of generalization could be sought by focusing on the fact that, in Experiment 2, the AN group was the only group in which stimulus A was presented in compound during both pre-exposure (An1, An2, An3…) and test (AX). Learning about the absence of consequences of the AN compounds during the pre-exposure could have been more readily generalized on test when another compound, AX, was presented. It could be argued that this would have led participants in the AN group to suppress their response more markedly. However, this type of explanation also faces notable problems. The first of these, again, is to explain the nature of the learning that supposedly is generalized. We would need to explain how this learning about the absence of consequences in the AN group is acquired, and that this explanation would indeed be an alternative to the one proposed by Hall and Rodríguez (2010). But, in addition, we would need to explain why similar learning did not occur, or was not expressed, in the condition in which A was exposed alone. It could be argued that in this condition, being pre-exposed to a stimulus (A) presented alone and tested with a compound (AX), there would be a certain decrease in generalization that would complicate the transfer of what was learned during pre-exposure. This argument is weakened, however, if we take into account some considerations regarding the notion of stimulus similarity and its relation to generalization and to our results.

The idea that, in Experiment 2, there was more generalization in the AN group than in the A group rests on the assumption that the compound cue weighed more heavily on stimulus similarity than the proportion of shared items itself (e.g., Estes, 1964; Pearce, 1987). The proportion of shared items is higher for group A (generalizing from A to AX) than for group AN (generalizing from multiple An to AX stimuli), so by this criterion, one would expect just the opposite of what is claimed, i.e., one would expect more generalization in group A than in group AN. This theoretically weakens the proposal but does not refute it. That is, there is no strong theoretical argument that denies the possibility that a composite cue could become so perceptually effective as to generate a decrease in generalization that reverses the effect of the proportion of items shared by the stimuli on generalization. More definitive, however, is the empirical refutation that occurs when this assumption is contrasted with our results from Experiment 1. In this experiment, if the compound cue had modulated the perception of stimulus similarity, a decrease in generalization of latent inhibition should have been observed in the AN group (which was pre-exposed to compounds but conditioned with an A stimulus presented alone) relative to the A group (which was pre-exposed and conditioned with the same A stimulus). The observation of the opposite pattern of results (more latent inhibition in the AN group than in the A group) suggests that the participants in our experiments did not exploit the compound cue as a particularly important determinant of stimulus similarity.

Ruling out generalization-based explanations, we might consider explaining the results in terms of external inhibition rather than conditioned inhibition per se. This would imply assuming that, in the AN group, a particularly salient A would have diminished the ability of × to evoke the response in the test, either by diverting attention or by interfering with the identification of × as the stimulus that had been previously conditioned. The problem with this explanation is that it would require assuming that A turned out to be more salient in the AN group, following its compound pre-exposure with novel stimuli, than in the NP group, where it was a novel stimulus in the test. In addition to the fact that it is difficult to imagine a mechanism that would be capable of generating this increase in the salience of A, the results of Experiment 1 clearly refute that this increase occurred. If A had gained salience during the pre-exposure of the AN group, a facilitation of excitatory learning would have been observed in this group instead of the particularly marked retardation that was observed.

In summary, therefore, explanations based on generalization and external inhibition do not provide a congruent framework to explain the results of our current and previous (Liberal et al., 2020, 2022) experiments.

Two pertinent issues arise at this point. The first is to specify how we account for the present and previous (Liberal et al., 2020, 2022) results from our theoretical perspective, and the second is what practical implications these results may have, with emphasis on the clinical domain.

We explain the results obtained through the latent inhibition model proposed by Hall and Rodríguez (2010, 2011). According to this model, novel stimuli are not completely neutral but their occurrence entails the associative activation of a, more or less, general expectation that something is going to happen. We locate the origin of this intrinsic activation in two possible mechanisms. We recognize that it may have an innate origin but emphasize that it can be generated and/or strengthened through experience. In the physical world in which we live, most events experienced by an organism are followed by some kind of consequence. And, furthermore, although of different kinds, these consequences also have certain common elements, the most basic and common to all consequences being that they are some kind of event. From these logical considerations, it can be expected that through a generalization mechanism, the appearance of a new stimulus will activate the expectation that something may occur through the elements in common that it has with other previously experienced stimuli. In other words, this analysis entails assuming that by learning different associations involving different specific stimuli, organisms continually will strengthen the acquisition of a general associative structure that helps predict that the appearance of any novel stimulus will may be followed by some sort of consequences. Although this associative structure is not confirmed on all occasions (i.e., some stimuli have no consequences), the high frequency of episodes in which it is confirmed will strengthen an excitatory association between the most basic and general representational node shared by all stimuli and the most basic and general representational node shared by all events that have constituted some kind of consequences in the past (see Rodríguez and Hall, 2017, for a further discussion in this matter).

The main implication of these considerations for explaining latent inhibition is that non-reinforced pre-exposure will become a case of extinction. That is, a novel stimulus will activate the expectation that something may happen, and when experience disconfirms this expectation, the association on which it is based will become progressively extinct. At a formal level, we place these assumptions in the Pavlovian conditioning model of Pearce and Hall (1980). According to this model, the attention a stimulus receives is inversely proportional to the uncertainty about its consequences. Hall and Rodríguez’s (2010) explanation of latent inhibition based on this model thus takes into account two different mechanisms. On the one hand, an attentional mechanism: during non-reinforced pre-exposure to the stimulus, the expectation that the stimulus will have consequences will be extinguished. Thus, the organism’s knowledge (the strength of the associations in which the stimulus is involved) will be adjusted to the environmental conditions experienced (the absence of consequences), which will produce a decrease in attention to the responsible stimulus capable of generating delay in any subsequent excitatory learning (i.e., the latent inhibition effect). The second mechanism would be an associative interference mechanism. According to our assumptions, a novel stimulus is not an associatively neutral stimulus. Novel stimuli will be associatively neutralized, progressively, during their non-reinforced pre-exposure. This will occur because, following the most accepted principles in the field of associative learning about how inhibitory properties are acquired (Pearce and Hall, 1980; Rescorla and Wagner, 1972), such pre-exposure will allow the stimulus to acquire a maximum inhibitory strength only similar to, but never greater than, the initial excitatory strength. That is, under normal conditions, a stimulus pre-exposed in the absence of consequences (i.e., a standard latent inhibitor) will have some inhibitory associative strength but no net inhibitory value. The acquisition of inhibitory strength will only compensate for the existence of some initial excitatory associative strength (which will not be erased or destroyed by inhibitory learning, e.g., Bouton, 1993; Westbrook and Bouton, 2010). This absence of a net inhibitory value may explain why under standard conditions (i.e., the presentation of a single stimulus alone in the absence of consequences), a latent inhibitor is unable to pass a summation test (e.g., Rescorla, 1969). However, according to these described mechanisms, the special pre-exposure schedule we have tested in the present work and in our previous work (Liberal et al., 2020, 2022), would indeed endow the target stimulus with net inhibitory properties. The presence of a novel stimulus in each pre-exposure trial alongside the target stimulus will ensure a higher-than-normal activation of the expectation that something is going to happen (i.e., by being generated by both the target stimulus and the novel stimulus). The target stimulus will gradually extinguish its initial excitatory strength but, in this case, the permanent presence of a different novel stimulus on each trial will ensure that the target can continue to acquire inhibitory strength beyond that set by its initial excitatory value, thus causing it to reach a net inhibitory value. Under these conditions, a stimulus can be expected to pass the summation test.

The present findings and their present explanation could have interesting implications for the clinical field. Latent inhibition is often considered as one of the factors that help explain individual differences in the acquisition of fear and the development of related anxiety disorders (Coelho et al., 2021; Mineka and Oehlberg, 2008; Mineka and Zinbarg, 2006; Pittig et al., 2018; Scheveneels et al., 2019). Specifically, the pre-exposure to a stimulus in the absence of consequences that characterizes latent inhibition is considered a protective factor for the subsequent development of phobias involving that stimulus (Coelho et al., 2021; Mineka and Oehlberg, 2008; Pittig et al., 2018; Scheveneels et al., 2019). For example, having a painful experience (US) in a dentist’s office (CS) will be less likely to result in the acquisition of a dental phobia (i.e., the occurrence of a fearful CR to this stimulus) if one has previously visited the dentist and has not had painful experiences (e.g., Kent, 1984). It is known that this protective effect of fear from latent inhibition can also be produced through vicarious experiences (e.g., Mineka and Cook, 1993), a phenomenon sometimes referred to as *fear immunization* (e.g., Coelho et al., 2021). The results of the experiments just presented (together with those of Liberal et al., 2020, 2022) suggest that one way to further enhance this protective effect on fear acquisition is to perform pre-exposure to the target stimulus together with different novel stimuli. This may also relate to the role of parenting in anxiety disorders. Several studies have shown that parents of anxious children are more likely to engage in more protective parenting (e.g., Rapee et al., 2009), which leads to the restriction of children’s exposure to varied and different situations. Our results and explanatory framework suggest a particular mechanism through which this lack of exposure to varied situations and stimuli may have a negative impact. If the most frequently occurring stimuli (equivalent to our target stimulus A) are not experienced in the presence of a variety of events (equivalent to our stimuli n1, n2, n3…), they will not become latent inhibitors with net inhibitory properties. That is, in the absence of experience with that variety of situations, the protection of latent inhibition will not be as effective as it could be and the possible negative experiences that a child, or any person in general, may suffer will more likely result in anxiety disorders based on exacerbated fear responses.

Finally, it is important to highlight the implications that the present results could also have for identifying potential inhibitors to the effectiveness of exposure therapies (Craske et al., 2014, 2022). In this case, we start from a problematic excitatory association between a CS (the phobic stimulus) and a fear-producing US. The goal of therapy is to extinguish this association through exposure to the CS in the absence of the US. The effectiveness of this type of therapy (i.e., the effectiveness of extinction trials) depends on the violation of expectancy, i.e., the perception of a discrepancy between the expected negative consequences (the occurrence of the US) and the actual absence of those consequences. The presence of conditioned inhibitors (i.e., safety signals) during exposure therapy leads to a reduction in the effectiveness with which the expectation of US occurrence is activated in the face of CS presentations, thus hindering the successful development of therapy. Our results and our explanatory framework point to a specific type of stimuli that may play this detrimental role but go unnoticed in the eyes of the therapist or the patient, as these stimuli do not appear to be conditioned inhibitors or safety signals due to their lack of explicit training. The situations that normally lead to a stimulus becoming a conditioned inhibitor involve the negatively correlated presentation of both the stimulus that will become inhibitor and the US. Our results, however, suggest that a stimulus can acquire some inhibitory properties without the need for its occurrence to have been experienced in the explicit absence of a US. We do not know, however, whether the inhibitory properties acquired by a latent inhibitor in the type of non-reinforced pre-exposure we have tested (An1, An2, An3…) are comparable to those of an explicitly trained safety signal in the absence of a specific US experienced in other nearby trials. As mentioned earlier, conditioned inhibitors seem to contain information about the specific outcome that is absent, while latent inhibitors operate on some more general characteristics assumed common to outcomes that constitute the expectation of an otherwise unspecified event. Given that characteristic, they may prove to be better at alleviating anxiety in general than the more specific conditioned inhibitor, even if their overall inhibitory power for a specific outcome is weaker.

## Data Availability

The raw data supporting the conclusions of this article will be made available by the authors, without undue reservation.
